# Endogenous mitochondrial hydrogen peroxide regulates neurogenesis during cortical development

**DOI:** 10.1016/j.redox.2025.103940

**Published:** 2025-11-19

**Authors:** Regina Mengual, Verónica Bobo-Jiménez, Cristina Rodríguez, Rebeca Lapresa, Darío García-Rodríguez, Daniel Jiménez-Blasco, Elisa Cabiscol, Joaquim Ros, María Delgado-Esteban, Juan P. Bolaños, Ángeles Almeida

**Affiliations:** a Instituto de Biología Funcional y Genómica, CSIC, Universidad de Salamanca, Salamanca, Spain; b Instituto de Investigación Biomédica de Salamanca, Hospital Universitario de Salamanca, Universidad de Salamanca, CSIC, Salamanca, Spain; c Centro de Investigación Biomédica en Red on Frailty and Healthy Ageing (CIBERFES), Madrid, Spain; d Dept. Ciències Mèdiques Bàsiques, Universitat de Lleida and IRBLleida, Lleida, Spain

**Keywords:** Mitochondria, Hydrogen peroxide, Neural precursor cells, Neurogenesis, Cortex development

## Abstract

Reactive oxygen species (ROS), particularly superoxide anion (O_2_^•-^) and hydrogen peroxide (H_2_O_2_), originating from mitochondria, are increasingly recognized as critical mediators of physiological signaling and cellular function. While in the adult brain, mitochondrial ROS, specifically mitochondrial H_2_O_2_, modulate metabolism and sustains cognitive processes, their role in the developing cerebral cortex remains undefined. Here, we leverage a knock-in mouse model constitutively expressing mitochondrially targeted catalase (mCAT) to attenuate mitochondrial H_2_O_2_ levels and investigate their impact during cortical development. In neurosphere cultures derived from embryonic day 14.5 (E14.5) mCAT mice, reduced mitochondrial H_2_O_2_ altered glutathione redox homeostasis and glucose metabolism leading to suppressed progenitor cell proliferation, without compromising viability. *In vivo*, neural progenitor cell (NPC) proliferation, neuronal differentiation and cortical layering were disrupted starting at gestational day E15. Together, these data uncover a physiological role for mitochondrial hydrogen peroxide in orchestrating neural precursor proliferation and differentiation, ultimately influencing mammalian cerebral cortex formation.

## 1 Introduction

Reactive oxygen species (ROS) had been considered merely toxic byproducts of metabolism, yet their role in physiological signaling, particularly from mitochondria, has become increasingly appreciated. Mitochondrial ROS contribute to metabolic adaptation under hypoxia and govern immune and inflammatory signaling pathways by acute redox signaling, while excessive ROS levels can activate cell death cascades such as apoptosis and autophagy [1,2]. In the adult brain, finely tuned mitochondrial ROS, particularly mitochondrial H_2_O_2_ generation in astrocytes, modulates neuronal metabolism, synaptic plasticity, and cognitive functions, including memory formation [3,4].

Outside the mature brain, mitochondria also significantly influence neural development [5,6]. The cerebral cortex is the most complex and evolutionarily advanced region of the brain, responsible for higher cognitive functions such as perception, reasoning, memory, and voluntary motor control. Its development is a highly coordinated process involving neurogenesis, neuronal migration, differentiation, and synaptogenesis, which collectively shape the intricate neural circuits necessary for cognitive and sensory functions [7]. Cortical development begins during early embryogenesis with the proliferation of neural progenitor cells (NPC) in the ventricular zone [8]. These progenitors generate excitatory and inhibitory neurons, which migrate radially and tangentially to establish the six-layered structure of the cortex. Once positioned, neurons differentiate, extend axons and dendrites, and form synaptic connections, guided by genetic programs and extracellular signaling cues [9,10].

It is known that mitochondrial integrity is essential for neural stem cells and NPC proliferation and fate decisions during corticogenesis [5], and mitochondrial dysfunction underlies both neonatal and adult NPC depletion, leading to impaired neurogenesis and neurodevelopmental deficits [11]. Interestingly, mitochondrial O_2_^•-^ intermittent bursts have been shown to negatively regulate NPC proliferation *in vitro*, and enhancing O_2_^•-^ by knocking out superoxide dismutase-2 (SOD2) mimicked the impaired NPC proliferation [12]. However, whether the concomitant decreased H_2_O_2_ in the SOD2 knockout cells might mediate this effect remained unclear. Notably, mitochondria-derived H_2_O_2_ appears to influence the balance between glycolysis and the pentose phosphate pathway (PPP) [3], impacting NADPH(H^+^) production and redox homeostasis, critical for proliferative demands and antioxidant defense. Yet, the *in vivo* role of mitochondrial H_2_O_2_ in governing NPC proliferation, metabolic flux, and cortical layering remains uncharted.

Here, we used a genetically-engineered mouse model expressing mitochondrially localized catalase (mCAT) to attenuate mitochondrial H_2_O_2_ *in vivo* and *in vitro*. We investigated the downstream effects on NPC proliferation, cortical layering, metabolic fluxes, and redox status. Our findings reveal that physiological H_2_O_2_ is indispensable in directing neurogenesis and cortical growth *via* integrated metabolic and redox-mediated mechanisms.

## 2 Results

**Fig. 1:**
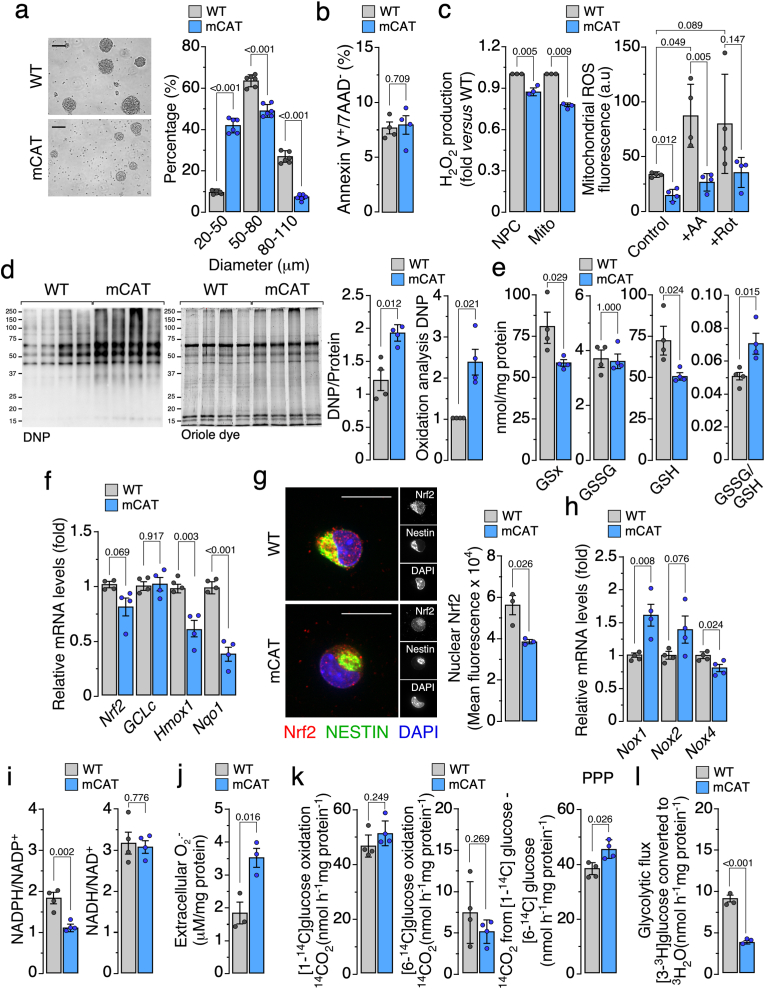
Mitochondrial H_2_O_2_ shapes redox signaling and glucose metabolism in embryonic neural precursors. **(a)** Representative bright-field pictures (left panel) of 6 days *in vitro* (6 DIV) primary neurospheres and size distribution plot showing the percentage of neurospheres distributed by diameter range (right panel). Scale bar: 80. Data are mean ± S.E.M. *P* value is indicated; n = 6 independent samples per genotype; Welch's *t*-test or unpaired, two-tailed Student's *t*-test *versus* WT. **(b)** Percentage of apoptotic cell death (Annexin V^+^/7-AAD^-^ cells) detected by flow cytometry. Data are mean ± S.E.M. *P* value is indicated; n = 4 independent samples per genotype; unpaired, two-tailed Student's *t*-test *versus* WT. **(c)** H_2_O_2_ production in whole-cell and mitochondrial fractions (left panel) isolated from 6 DIV neurospheres detected by fluorimetry (fold change *versus* corresponding WT value). Mitochondrial ROS fluorescence (right panel) quantified by flow cytometry under basal (control) and pharmacological conditions (1 μM Rot: Rotenone; 1 μM AA: Antimycin A). Data are mean ± S.E.M. *P* value is indicated. Left panel: n = 3 independent samples per genotype; unpaired, two-tailed Student's *t*-test *versus* WT. Right panel: n = 4 independent samples per genotype; Kruskal-Wallis followed by Dunn's test correction. **(d)** Representative images showing the formation of protein oxidation (total carbonyl groups) and detection by anti-DNP (2,4-dinitrophenol) antibody (Oxyblot) and total-protein loading control detected by oriole dye staining (left panel). Band intensities were quantified by densitometry (right panel). Data are mean ± S.E.M. *P* value is indicated; n = 4 independent samples per genotype; Welch's *t*-test *versus* WT. **(e)** Concentrations of total (GSx), oxidized (GSSG), and reduced (GSH) glutathione and GSSG/GSH ratio in 6 DIV neurospheres. Data are mean ± S.E.M. *P* value is indicated; n = 4 biologically independent samples per genotype; unpaired, two-tailed Student's *t*-test or unpaired U-Mann-Whitney's test *versus* WT. **(f)** Relative mRNA levels of *Nrf2* and its target (*GCLc*, glutamate-cysteine ligase, catalytic subunit; *Hmox1*, heme oxygenase 1; Nqo1, NAD(P)H:quinone oxidoreductase 1) genes. Data are mean ± S.E.M. *P* value is indicated; n = 4 independent samples per genotype; unpaired, two-tailed Student's *t*-test *versus* WT. **(g)** Representative images of Nrf2 (red) and NESTIN (green) immunofluorescence staining (left panel) and quantifications of the mean intensity of nuclear Nrf2 fluorescence (right panel). Scale bar: 5 μm. Data are mean ± S.E.M. *P* value is indicated; n = 3 independent samples per genotype; unpaired, two-tailed Student's *t*-test *versus* WT. **(h)** Relative mRNA levels of Nox isoforms. Data are mean ± S.E.M. *P* value is indicated; n = 4 independent samples per genotype; unpaired, two-tailed Student's *t*-test *versus* WT. **(i)** NADPH(H^+^)/NADP^+^ and NADH(H^+^)/NAD^+^ ratios measured in 6 DIV neurospheres. Data are mean ± S.E.M. *P* value is indicated; n = 4 independent samples per genotype; unpaired, two-tailed Student's *t*-test *versus* WT. **(j)** Extracellular superoxide produced by 6 DIV neurospheres. Data are mean ± S.E.M. *P* value is indicated; n = 3 independent samples per genotype; unpaired, two-tailed Student's *t*-test *versus* WT. **(k)** Rates of ^14^CO_2_ production from [1–^14^C]-glucose (left) and [6–^14^C]-glucose (middle) in 6 DIV neurospheres. Right panel shows the difference between the rates of ^14^CO_2_ production from [1–^14^C]- and [6–^14^C]-glucose, indicating the rate of glucose oxidation *via* the PPP (pentose phosphate pathway). Data are mean ± S.E.M. *P* value is indicated; n = 4 independent samples per genotype; unpaired, two-tailed Student's *t*-test *versus* WT. **(l)** Glycolytic flux measured by ^3^H_2_O release from [3-^3^H]-glucose in neurospheres. Data are mean ± S.E.M. *P* value is indicated; n = 3 independent samples per genotype; unpaired, two-tailed Student's *t*-test *versus* WT.

To investigate whether mitochondrial H_2_O_2_ plays a functional role in corticogenesis, we employed a knock-in mouse model expressing mitochondrially targeted catalase (mCAT), which selectively attenuates mitochondrial H_2_O_2_ [3,4]. We first characterized *in vitro* consequences of mCAT expression in neural precursors and subsequently assessed *in vivo* effects on cortical development. We derived neurospheres from embryonic day 14.5 (E14.5) cortices of mCAT and wild-type (WT) mice. As shown in Fig. 1a, neurospheres formed by mCAT NPC were visibly smaller, suggesting altered proliferative and/or survival capacity. Importantly, cell viability remained comparable between genotypes (Fig. 1b), indicating that increased cell death did not account for sphere size differences. Biochemical analyses revealed that mitochondrial H_2_O_2_ production was selectively reduced in mCAT neurosphere cultures (Fig. 1c), with a corresponding decrease in mitochondrial ROS fluorescence under both basal and pharmacologically challenged conditions. Surprisingly, global protein oxidation was higher in mCAT NPC, as shown by OxyBlot (Fig. 1d), while total protein loading was verified by Oriole staining. Redox profiling further demonstrated that glutathione homeostasis was reconfigured, with alterations in reduced (GSH) and oxidized (GSSG) pools and a shift in the GSSG/GSH ratio (Fig. 1e). Correspondingly, transcripts of the nuclear factor erythroid 2-related factor 2 (Nrf2) antioxidant program -including Nrf2 itself and its potent downstream targets, *heme oxygenase 1 (Hmox1) and NAD(P)H:quinone oxidoreductase* (*Nqo1)* [13]- were modulated, suggesting attenuated redox signaling in mCAT NPC (Fig. 1f). However, gene expression of the Nrf2 target *glutamate-cysteine ligase, catalytic subunit (GCLc),* maintained unaffected, suggesting a compensatory positive regulation by other antioxidant-responsive transcription factors, such as AP-1, NF-κB or SP1 [[14], [15], [16]]. Immunofluorescence indicated reduced nuclear localization of Nrf2 in mCAT NPC (Fig. 1g), corroborating suppressed pathway activation. Cross-talk with membrane NADPH(H^+^) oxidase systems was evident. Thus, expression of NADPH oxidase (Nox) isoforms (Fig. 1h), Nox1 and Nox2, were increased, whereas Nox 4 decreased, in mCAT NPC. Moreover, NADPH(H^+^)/NADP^+^ decreased while NADH(H^+^)/NAD^+^ ratio remained unchanged in mCAT NPC (Fig. 1i). We next analyzed extracellular O_2_^•-^ abundance in neurosphere cultures as a measure NOX activity [3,4]. In agreement with Nox-1 and Nox-2 upregulation, extracellular O_2_^•-^ generation by mCAT neurosphere was enhanced (Fig. 1j). Finally, metabolic flux assays revealed differential routing of glucose; thus, while complete oxidation of glucose *via* the TCA cycle was reduced, the pentose phosphate pathway increased (Fig. 1k); this was accompanied by a concomitant decrease in the glycolytic flux in mCAT neurospheres (Fig. 1l). Together, these data demonstrate that mitochondrial H_2_O_2_ acts as an important redox-metabolic integrator in NPC, shaping antioxidant signaling and redirecting glucose metabolism without compromising cell survival.

**Fig. 2:**
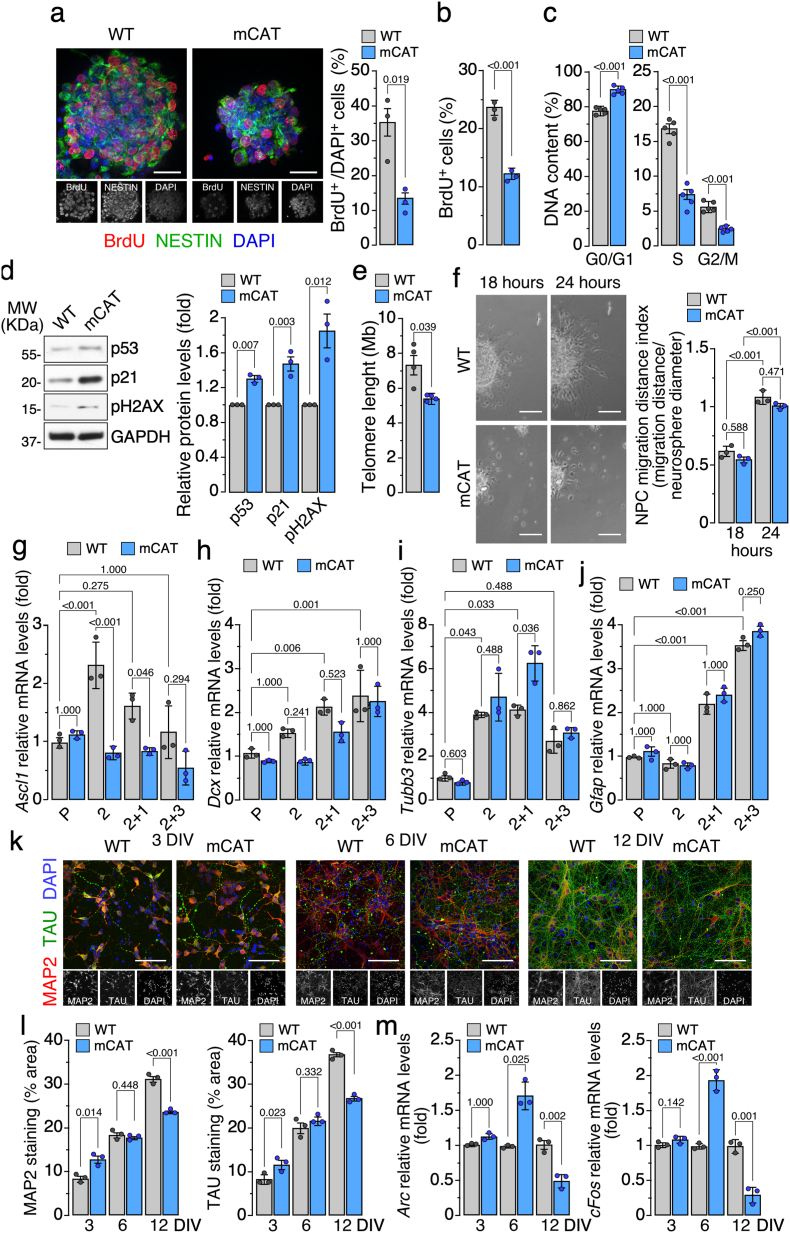
Reduced mitochondrial H_2_O_2_ limits progenitor proliferation and neuronal differentiation *in vitro*. **(a)** Representative images of BrdU (red) and NESTIN (green) immunofluorescence staining (left panel) and percentage of BrdU^+^/DAPI^+^ NPC (right panel). Scale bar: 40 μm. Data are mean ± S.E.M. *P* value is indicated; n = 3 independent samples per genotype; unpaired, two-tailed Student's *t*-test *versus* WT. **(b)** Percentage of BrdU^+^ NPC quantified by flow cytometry. Data are mean ± S.E.M. *P* value is indicated; n = 3 biologically independent samples per genotype; unpaired, two-tailed Student's *t*-test *versus* WT. **(c)** Distribution of cell-cycle phases as determined by DNA-content analysis using flow cytometry. Data are mean ± S.E.M. *P* value is indicated; n = 5 independent samples per genotype; unpaired, two-tailed Student's *t*-test *versus* WT. **(d)** Representative western blots and quantification of protein levels of p53, p21, and γH2AX, with loading control GAPDH. Data are mean ± S.E.M. *P* value is indicated; n = 3 independent samples per genotype; Welch's *t*-test or unpaired, two-tailed Student's *t*-test *versus* WT. **(e)** Telomere length measurement. Data are mean ± S.E.M. *P* value is indicated; n = 4 independent samples per genotype; Welch's *t*-test *versus* WT. **(f)** Primary neurospheres were plated on poly-d-lysine- and fibronectin-coated glass coverslips in mitogen-free media. Phase-contrast images of NPC migration out from neurosphere (left panel), at 18 h and 24 h after plating. Quantifications of NPC migration were expressed as the migration distance index (NPC radial migration distance (μm)/neurosphere diameter (μm) (right panel). Scale bar: 40 μm. Data are mean ± S.E.M. *P* value is indicated; n = 3 independent samples per genotype; one-way ANOVA followed by Bonferroni correction. **(g**–**j)** Relative mRNA levels of *Ascl1*, *Dcx*, *Tubb3*, and *Gfap* at indicated condition/days (P: neurospheres cultured in proliferation medium for 6 DIV; 2: NPC cultured in DMEM/F12 medium supplemented with bFGF (10 ng/ml) for 2 days; 2 + 1 and 2 + 3: cells cultured in DMEM/F12 medium supplemented with 2 % fetal bovine serum for 1 and 3 days after bFGF withdrawal). Data are mean ± S.E.M. *P* value is indicated; n = 3 independent samples per genotype; (**g** and **j**) one-way ANOVA followed by Bonferroni correction; (**h**) Welch's ANOVA test followed by Games-Howell correction and (**i**) Kruskal-Wallis followed by Dunn's test. **(k)** Representative images of primary cortical neurons immunostained with MAP2 (red) and TAU (green) at 3, 6, and 12 DIV. Nuclei were stained with DAPI (blue). Scale bar: 100 μm **(l)** Quantification of MAP2 and TAU immunostaining (% area) in primary cortical neurons, at 3, 6, and 12 DIV. Data are mean ± S.E.M. *P* value is indicated; n = 3 independent samples per genotype; unpaired, two-tailed Student's *t*-test *versus* WT. **(m)** Relative mRNA levels of *Arc* and *cFos* in primary cortical neurons, at 3, 6, and 12 DIV. Data are mean ± S.E.M. *P* value is indicated; n = 3 independent samples per genotype; unpaired, two-tailed Student's *t*-test or Welch's *t*-test *versus* WT.

Given the metabolic shifts observed, we next assessed functional consequences on proliferation and neuronal differentiation. Bromodeoxyuridine (BrdU) incorporation assays revealed a marked reduction in S-phase entry among mCAT NPC (Fig. 2a and b). Cell-cycle profiling confirmed accumulation in G_0_/G_1_ phase with concomitant depletion of S and G_2_/M populations (Fig. 2c). Immunoblots showed increased levels of cell-cycle checkpoint and oxidative DNA damage markers -including p53, p21, and γ phosphorylated form of the histone H2AX (γH2AX)- (Fig. 2d) and the consequent telomere length shortening (Fig. 2e) in mCAT cultures, likely driven by oxidative DNA damage [17], all indicating a senescence-dependent mechanisms [18], underpinning the proliferative defect.

At the light of the cell cycle impairments observed in the mCAT NPC, we next aimed to ascertain its impact on the process of neuronal differentiation. As shown in Fig. 2f, attenuation of mitochondrial H_2_O_2_ did not alter the natural increase in radial migration from the neurosphere edges under proliferative culture conditions. However, following growth factor withdrawal, loss of mitochondrial H_2_O_2_ impaired the transient induction of the proneuronal transcription factor gene *Achaete-scute homolog 1* (*Ascl1*) [19] (Fig. 2g), decreased *Doublecortin* (*Dcx*, newborn neurons) [20] mRNA levels (Fig. 2h), and increased the cytoskeletal *Tubulin beta 3 class III* (*Tubb3*, encoding neuronal marker TUJ1) [21] gene expression (Fig. 2i) indicating an accelerated progression towards the neuronal differentiated state. However, the astrocytic marker *Glial fibrillary acidic protein* (*Gfap*) [22] remained unchanged in the mCAT NPC (Fig. 2j). Thus, loss of H_2_O_2_ in mitochondria does not affect glial differentiation, but rather the neuronal differentiation process itself. To directly test this issue, we measured the protein levels of the maturation neuronal markers Microtubule-associated protein 2 (MAP2) and TAU by immunocytochemistry. Decreased mitochondrial H_2_O_2_ increased the protein abundances of these proteins at early time points (3–6 days), indicating premature differentiation process (Fig. 2k and l). However, upon further incubation for a longer period (12 days) the protein levels of MAP2 and TAU decreased in the mCAT (Fig. 2k and l), reflecting neuronal loss or neurite disruption. Interestingly, the activity-dependent immediate-early genes *Arc* (activity-regulated cytoskeleton-associated protein) and *cFos* -markers of activity of fully differentiated neurons [23]-, increased at 6 DIV in mCAT neurons (Fig. 2m); however, it was decreased at 12 DIV (Fig. 2m), echoing MAP2 and TAU loss, which indicates neuronal dysfunction and degeneration [23]. These findings suggest that lowering mitochondrial H_2_O_2_ in neural precursors hampers neural precursor cells proliferation and prematurely accelerates their differentiation into neurons, finally ending in a loss of function.

**Fig. 3:**
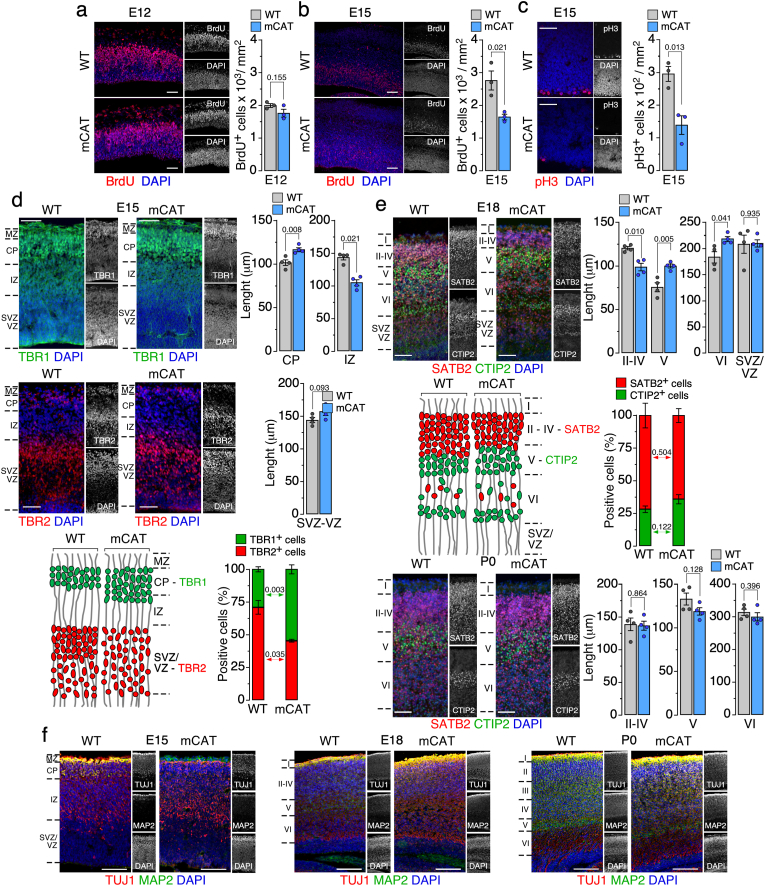
Mitochondrial H_2_O_2_ is required *in vivo* for progenitor proliferation and proper cortical lamination. (a) Representative images of BrdU labeling at embryonic day 12 (E12) (left panel) and quantification of BrdU^+^ cells per area (right panel). Scale bar: 50 μm. Data are mean ± S.E.M. *P* value is indicated; n = 3 mice per genotype; unpaired, two-tailed Student's *t*-test *versus* age-matched WT. **(b)** Representative images of BrdU labeling at embryonic day 15 (E15) (left panel) and quantification of BrdU^+^ cells per area (right panel). Scale bar: 100 μm. Data are mean ± S.E.M. *P* value is indicated; n = 3 mice per genotype; unpaired, two-tailed Student's *t*-test *versus* age-matched WT. **(c)** Representative images of mitotic marker, pH3, immunostaining at E15 (left panel) and mitotic cell density (pH3^+^ cells/area) quantification (right panel). Scale bar: 50 μm. Data are mean ± S.E.M. *P* value is indicated; n = 3 mice per genotype; unpaired, two-tailed Student's *t*-test *versus* age-matched WT. **(d)** Representative images of E15 immunostaining for TBR1 (deep-layer neurons; green) and TBR2 (intermediate progenitors; red) with corresponding measurements of cortical plate (CP), intermediate zone (IZ) (*upper panels*) and subventricular-ventricular zones (SVZ-VZ) (*middle panels*) lengths. Scheme representing TBR1^+^ (green) and TBR2^+^ (red) cell distribution from immunofluorescence images and quantification of the proportion of positive cells (*lower panel*). TBR1 scale bar: 100 μm. TBR2 scale bar: 50 μm. Data are mean ± S.E.M. *P* value is indicated; n = 4 mice per genotype in *upper* and *middle* panels, and n = 3 mice per genotype in *lower* panel; unpaired, two-tailed Student's *t*-test; unpaired U-Mann-Whitney's test or Welch's *t*-test *versus* age-matched WT. **(e)** Representative images of E18 immunostaining for CTIP2 (layer V; green) and SATB2 (layers II-IV; red) and with corresponding measurements of layer II-IV, V, VI and SVZ-IV lengths (*upper panels*). Scheme representing CTIP2^+^ (green) and SATB2^+^ (red) cell distribution from immunofluorescence images and quantification of the proportion of positive cells (*middle panel*). P0 staining for CTIP2 (green) and SATB2 (red) with measurements of layer II–IV, V and VI lengths (*lower panels*). Scale bar: 100 μm. Data are mean ± S.E.M. P value is indicated. n = 4 mice per genotype; Welch's *t*-test or unpaired, two-tailed Student's *t*-test *versus* age-matched WT. **(f)** Immunostaining for neuronal markers, TUJ1(red) and MAP2 (green), at E15, E18, and P0. Scale bar: 100 μm.

We then examined whether the *in vitro* phenotypes translated to *in vivo* developmental outcomes. At E12, BrdU labeling revealed no significant differences in progenitor proliferation between WT and mCAT cortices (Fig. 3a), indicating intact early cell proliferation. By E15, however, BrdU incorporation was significantly reduced in mCAT embryos (Fig. 3b), and mitotic activity, assessed *via* phospho-histone 3 (pH3, mitotic marker) staining, was diminished (Fig. 3c), demonstrating an emerging proliferation deficit during mid-gestation. Immunostaining for deep-layer neuron marker TBR1 (T-box brain transcription factor 1) and intermediate progenitor marker TBR2 (T-box brain transcription factor 2) [24] revealed alterations in the cortical zone organization at E15 (Fig. 3d), including enhanced cortical plate and decreased intermediate zone extents (*upper panels*), while the subventricular-ventricular zone length was unaltered (*middle panels*). These observations indicate that at E15, the reduction in mitochondrial H_2_O_2_ enhanced the proportion of deep layer *versus* the intermediate progenitor ones (*lower panels*). At E18, the laminar architecture was further perturbed. Thus, increased Special AT-rich sequence-binding protein 2 (SATB2; layers II-IV [25]) and decreased B-cell leukaemia/lymphoma 11B (CTIP2, also known as BCL11b; layer V [26]) staining indicated a shift in the relative sizes of these cortical layers, without major changes in the subventricular-ventricular zones (Fig. 3e*, upper panels*). Whilst these findings demonstrate changes in the organization of the cortical zone, the proportion of cells were not altered (*middle panels*). These alterations took place during late gestation, since no alterations were observed at P0 (*lower panels*), when neuronal migration and six-layered structure become established [27]. This developmental trajectory was confirmed by the analysis of TUJ1 and MAP2 from E15 through P0, which visually revealed the cortical layers disorganization in the mCAT mice (Fig. 3f). These *in vivo* findings underscore a requirement for mitochondrial H_2_O_2_ in sustaining late-embryonic progenitor proliferation and orchestrating appropriate neuronal differentiation, thereby ensuring correct cortical lamination.

## 3 Discussion

In sum, our *in vitro* findings revealed that the selective attenuation of mitochondrial H_2_O_2_ disrupts redox homeostasis -dampening Nrf2 pathway activation, altering glutathione pools, and rewiring glucose metabolism toward increased PPP and reduced glycolytic flux. These observations echo emerging insights into mitochondrial dynamics during neurodevelopment. Thus, mitochondrial ROS can trigger Nrf2 driven transcriptional programs that influence neural progenitor fate, e.g., *via* fission fusion dynamics -a concept illustrated by developmental shifts in mitochondrial morphology influencing redox signaling and differentiation [5]. Our data extend this paradigm, showing that H_2_O_2_ itself, independent of overt mitochondrial morphological changes, functions as a core redox‐metabolic signal directing NPC physiology.

Prior studies have shown that mitochondrial O_2_^•-^ bursts inhibit NPC proliferation *in vitro*, implicating elevated mitochondrial ROS in suppressing division [12]. Our findings refine this model by showing that attenuated mitochondrial H_2_O_2_ -not elevated O_2_^•-^- is similarly detrimental; thus, reducing H_2_O_2_ also impairs proliferation, likely by reducing redox signaling necessary for cell‐cycle progression. Thus, both extremes of mitochondrial ROS signaling -excess or deficiency-can suppress proliferation, underscoring the need for finely tuned ROS dynamics in NPC fate decisions. Our data therefore underscore a dualistic role of ROS in neurodevelopment. At physiological levels, mitochondrial H_2_O_2_ functions as a signaling metabolite that coordinates redox state, metabolic adaptation, and progenitor behavior. Conversely, impairing H_2_O_2_ generation compromises developmental programs.

Cerebral cortex size and function depend on the coordination between NPC proliferation and the onset of neurogenesis, which ensures the correct neuron generation [9,28]. Decreased cell proliferation was observed in the cortex of mCAT mice *in vivo* at mid-late gestation, E15, but not at earlier E12, when NPC switch to a neurogenic proliferation and neurogenesis starts [29,30], then explaining the absence of microcephaly in the mCAT mouse. However, we found accelerated neurogenesis and altered cortical lamination patterns at late stages of development. Then, as neurogenesis advances, oxidative stress gains greater importance, becoming especially relevant at late gestation. It is possible that alterations found in the cortex of mCAT mice have long-term effects, leading to cognitive deficits in the adult, as it was observed when reducing mitochondrial H_2_O_2_ in astrocytes [3,4]. In this context, unproper neuronal cell type position and impaired lamination patterns are involved in the pathogenesis of autism and other neurodevelopmental disorders [30].

Several questions remain. First, while we show that attenuating mitochondrial H_2_O_2_ impairs proliferation and layering, the precise downstream effectors -such as specific redox-sensitive transcription factors or metabolic sensors-remain to be identified. We observed Nrf2 activation was blunted in mCAT cultures, implicating this pathway, but further mechanistic dissection is needed. In line with this, we also found that Nox1 and Nox2 expression were significantly increased in mCAT NPCs. Given that Nrf2 is known to suppress Nox isoforms, the reduced Nrf2 activity in mCAT is consistent with their upregulation. This dysregulation of NADPH oxidases provides a mechanistic explanation for the observed rise in extracellular O_2_^•-^ and the paradoxical increase in oxidative stress despite attenuated mitochondrial H_2_O_2_. These results are fully consistent with our previous observations in astrocytes [3], where Nrf2 deficiency was likewise associated with enhanced Nox activity and O_2_^•-^ production. Thus, rather than alleviating redox stress, the loss of H_2_O_2_ signaling impairs Nrf2-dependent antioxidant defenses and generates Nox-derived ROS, reinforcing the concept that physiological mitochondrial H_2_O_2_ is protective because it sustains Nrf2 activity and restrains Nox upregulation. Second, while our model focused on H_2_O_2_, it is important to understand how other ROS species -such as superoxide- and their interconversion dynamics influence neural development. Indeed, perturbing mitochondrial superoxide *via* SOD2 manipulations may simultaneously alter H_2_O_2_, with mixed effects on NPC biology [12]. Lastly, though our study emphasizes developmental roles, potential long-term consequences of mitochondrial H_2_O_2_ attenuation -for example in neural circuit formation, synaptic maturation, and adult cognitive functions-remain unexplored. Given the known roles of ROS signaling in adult synaptic plasticity such as, e.g., astrocytic H_2_O_2_ modulating memory formation [3], a developmental deficit in ROS signaling may have lasting behavioral outcomes.

In conclusion, we unveil a previously unappreciated role for mitochondrial H_2_O_2_ as a physiological redox-metabolic signal that orchestrates progenitor proliferation and cortical lamination during corticogenesis. Moreover, our findings provide *in vivo* relevance to the redox-metabolic shifts observed *in vitro*, indicating that the developmental effects of H_2_O_2_ attenuation are likely mediated by these integrated signaling and metabolic disturbances. These findings challenge the traditional conception of ROS solely as damaging by-products. Instead, they highlight finely balanced mitochondrial redox dynamics as critical for normal brain development. Future work exploring how ROS interface with metabolic and transcriptional networks promises to deepen our understanding of neurodevelopmental disorders and reveal novel targets for intervention.

## 4 Materials and methods

*Experimental model.* All animal procedures we performed according to the European Union Directive 2010/63/EU, 86/609/EEC and Recommendation 2007/526/EC, regarding the protection of animals used for experimental and other scientific purposes, enforced in Spanish legislation under the directive RD53/2013 y ECC/566/2015 and its modification CNU/120/2025. All protocols were approved by the Bioethics Committee of the University of Salamanca. A transgenic mouse harboring the full-length cDNA encoding catalase fused to the cytochrome *c* oxidase subunit VIII-mitochondrial leading sequence, and to the human influenza haemagglutinin for tagging purposes (mitochondrial-tagged catalase) was generated by homologous recombination in the Rosa26 locus under a C57BL/6 background [3]. Heterozygous constitutive mCAT mice were backcrossed with C57BL/6-J mice to perform *in vitro* and *in vivo* experiments with mCAT and wild-type (+/+ or WT) littermates. For BrdU incorporation analysis, pregnant mice were administered BrdU (50 mg/kg body weight) by intraperitoneal injection [28].

*Genotyping by polymerase chain reaction (PCR).* The primer sequences for genotyping the mCAT allele were 5′ -CTCCCAAAGTCGCTCTGAGTTGTTATCA-3′,5′ -CGATTTGTGGTGTATGTAACTAATCTGTCTGG-3′ and 5′GCAGTGAGAAGAGTACCACCATGAGTCC-3′, which yielded a 778-bp band for the wild-type allele and a 245-bp band for the mCAT allele [3].

*Cell cultures.* Primary cultures of mouse cortical neurons were prepared from E14.5 mCAT and WT mice, seeded at 180,000 cell/cm^2^ in different size plastic plates coated with poly-d-lysine (10 μg/ml), poly-l-ornitine (15 μg/ml) or fibronectine (1 μg/ml) and incubated in Neurobasal A medium (Invitrogen, Thermo Fisher Scientific) supplemented with 2 % B27-MAO (Minus AntiOxidants, Invitrogen), 2 mM glutamine, 5.5 mM d-glucose, 2.5 mM pyruvate (Invitrogen), and antibiotics (100 U/L penicillin G, 100 μg/L streptomycin, 0.25 μg/L amphotericin B; Sigma-Aldrich). Cells were incubated at 37 °C in a humidified 5 % CO_2_-containing atmosphere and recollected at different days *in vitro* (DIV) [28].

Neurosphere cultures were prepared from E14.5 mice mCAT and WT mice, seeded at 20000 cells/cm^2^ in different size plastic plates without coating. Cells was incubated in DMEM/F12 (17.5 mM glucose, Life Technologies, Thermo Fisher Scientific), supplemented with 2 % B27 (without vitamin A), 10 mM l-glutamine, 10 mM pyruvate, N2 supplement (Life Technologies), antibiotics (100 U/L penicillin G, 100 μg/L streptomycin, 0.25 μg/L amphotericin B; Sigma-Aldrich), and mitogens, 20 ng/ml bFGF (basic Fibroblast Growth Factor, Sigma) and 20 ng/ml EGF (Epidermal Growth Factor, Gibco Life Technologies) [31]. Neurosphere cultures were maintained at 37 °C with a humidified atmosphere containing 5 % CO_2_, for 6 days.

*Mitochondrial isolation.* Neurospheres were collected and homogenized in a glass-Teflon Potter-Elvehjem homogenizer in buffer A (83 mM sucrose and 10 mM MOPS, pH 7.2). The same volume of buffer B (250 mM sucrose and 30 mM MOPS; pH 7.2) was added to the sample, and the homogenate was centrifuged (1000×*g*, 5 min). The supernatant was then centrifuged (12,000×*g*, 2 min) to obtain the mitochondrial fraction, which was washed with buffer C (320 mM sucrose, 1 mM EDTA and 10 mM Tris-HCl, pH 7.4). Mitochondria were suspended in buffer D (1 M 6-aminohexanoic acid and 50 mM Bis-Tris-HCl; pH 7.0) [3].

*Mitochondrial ROS measurement.* Mitochondrial ROS was determined using the fluorescent MitoSox™ probe (Life Technologies), as previously done [3]. Neurospheres were incubated with 2 μM of MitoSox™ (Invitrogen) for 30 min at 37 °C in a 5 % CO_2_ atmosphere in Hanks' Balanced Salt Solution (HBSS) buffer (134.2 mM NaCl, 5.26 mM KCl, 0.43 mM KH_2_PO_4_, 4.09 mM NaHCO_3_, 0.33 mM Na_2_HPO_4_·2H_2_O, 5.44 mM glucose, 20 mM HEPES, 4 mM CaCl_2_·2H_2_O; pH 7.4). Neurospheres were collected, washed with phosphate-buffered saline (PBS; 0.1 M) and dissociated with Accutase™ (Stemcell Technologies). MitoSox fluorescence intensity was assessed by flow cytometry (FACScalibur flow cytometer, BD Biosciences) and expressed in arbitrary units.

*H*_*2*_*O*_*2*_ *determination.* For H_2_O_2_ assessments, an Amplex™ Red Hydrogen Peroxide/Peroxidase Assay Kit (Life Technologies) was used. Neurospheres at 6 DIV were dissociated with Accutase™ and subsequently resuspended in Krebs-Ringer phosphate buffer (KRPG) (145 mM NaCl, 5.7 mM Na_2_HPO_4_, 4.86 mM KCl, 0.54 mM CaCl_2_, 1.22 mM MgSO_4_ and 5.5 mM glucose, pH 7.4) containing 9.45 μM Amplex™ Red and 0.1 U ml^−1^ horseradish peroxidase. Luminescence was recorded for 2 h at 30-min intervals with a Varioskan Flash (Thermo Scientific) (λ excitation = 538 nm; λ emission = 604 nm). Slopes were used for the calculations of the rates of H_2_O_2_ formation, and the results were expressed as fold change versus the corresponding WT values [3].

*Extracellular superoxide (O2*^*•-*^*) determination.* Neurospheres were attached in 96-well plates, washed twice with KRPG and incubated in the presence of 120 μM of oxidized cytochrome *c* (Sigma-Aldrich) for 4 h at 37 °C. The absorbance at 550 nm was recorded, and O_2_^•-^ concentration calculated with the extinction coefficient of the reduced form of cytochrome *c* and normalized to protein concentration.

*Determination of protein oxidation.* Neurospheres were dissociated and collected in a RIPA buffer (1 % SDS, 10 mM EDTA, 1 % Triton Tx-100, 150 mM NaCl, 10 mM Na_2_PO_4_ at pH 7.0) supplemented with 2 % SDS. To this protein extract, 10 mM DNPH (2,4-dinitrophenylhydrazine) was added, and the mixture was heated for 10 min, at 25 °C. Subsequently, 1 M Tris, 30 % glycerol and 15 % β-mercaptoethanol was added to the sample. Finally, a conventional western blotting analysis was performed to detect 2,4-dinitrophenol (DNP).

*Western blotting.* Cells were lysed in RIPA buffer, supplemented with phosphatases inhibitors (1 mM Na_3_VO_4_, 50 mM NaF) and proteases inhibitors (100 μM phenylmethylsulfonyl fluoride, 50 μg/ml antipapain, 50 μg/ml pepstatin, 50 μg/ml amastatin, 50 μg/ml leupeptin, 50 μg/ml bestatin, 1 mM o-vanadate, 50 mM NaF, 50 μg/ml soybean trypsin inhibitor), stored on ice for 30 min, boiled for 5 min and sonicated for 10 min. Protein concentrations were measured with BCA (bicinchoninic acid) method, using BSA as a standard (BCA Protein Assay kit, Thermo Fisher Scientific #23225). Lysates (neurons and neurospheres) were submitted to SDS/PAGE on 6–12 % acrylamide gel (Mini-PROTEAN; Bio-Rad) including PageRulerTM Plus Prestained Protein Ladder (Thermo Fisher Scientific #26619). The primary antibodies used were anti-p21 (1:500; BD, #556431), anti-p53 (1:500, Cell Signaling, #2524) and anti-H2AX (1:500; Millipore; #05–63609). GAPDH (glyceraldehyde-3-phosphate dehydrogenase) was used as loading control. After incubation with horseradish peroxidase conjugated to secondary antibodies goat anti-mouse IgG (Bio-Rad #170–6516) or goat anti-rabbit IgG (Bio-Rad #170–6515), membranes were incubated with Pierce ECL Plus Western Blotting Substrate chemiluminescence kit (Thermo Fisher Scientific #32132), Super Signal West Femto Maximum Sensitivity Substrate (Thermo Fisher Scientific #34096) or Western Blotting Luminol Reagent (Santa Cruz Biotechnology #sc-2048), before exposure to Fuji Medical X-Ray film (Fujifilm), and the autoradiograms were then scanned. Protein abundance was measured by densitometry using ImageJ 1.48u4 software (National Institutes of Health, NIH) and were normalized per the loading control protein [28].

*Total, reduced and oxidized glutathione measurement.* Neurospheres were lysed with 1 % sulfosalicylic acid and centrifuged at 13,000×*g* for 5 min at 4 °C. Supernatants were used for the determination of total glutathione (reduced glutathione concentration plus twice the concentration of oxidized glutathione), using oxidized glutathione (GSSG; 0–50 μM) as a standard [32]. Total glutathione was measured in reaction buffer (0.1 mM Na_2_HPO_4_, 1 mM EDTA, 0.3 mM DTNB, 0.4 mM NADPH and glutathione reductase at 1 U ml^−1^, pH 7.5) by recording the increase in the absorbance at 405 nm after the reaction of reduced glutathione with 5,5′-dithiobis (2-nitrobenzoic acid) for 2.5 min at 15-s intervals using a Varioskan® Flash (Thermo Fisher). GSSG was quantified after derivatization of GSH with 2-vinylpyridine, by using similarly treated GSSG standards (0–5 μM), and the results are expressed as the oxidized glutathione/reduced glutathione ratio (GSSG/GSH).

*Real-time quantitative polymerase chain reaction (RT-qPCR).* Total RNA was purified from neurospheres using GenElute Mammalian Total RNA Miniprep Kit™ (Sigma-Aldrich), according to the manufacturer's protocol. Amplifications were performed in 100 ng of RNA, using Power SYBR Green RNA-to-CT^TM^1-step kit (Applied Biosystems). Forward and reserve primers were, respectively (Thermo Fisher Scientific), 5ʹ-GCAGGACATGGATTTGATTGA-3ʹ and 5ʹ-GTCAAACACTTCTCGACTTAC-3ʹ (*Nrf2*), 5′-GGCACAAGGACGTGCTCAAGT-3′ and 5′- TTTGTCCTCTCCCCCTTCTC-3’ (*Gcl)*, 5′-AGCACAGGGTGACAGAAGAG-3′ and 5′-GAGGGACTCTGGTCTTTGTG-3’ (*Hmox1*), 5ʹ-GGGGACATGAACGTCATTCTCT-3ʹ and 5ʹ-AAGACCTGGAAGCCACAGAAGC-3ʹ (*Nqo1*), 5′-AAATTCCAGCGTGCCGACAA-3′ and 5′-AACCAGGCAAAGGCACCTGT-3' (*Nox1*), 5′-ATGCAGGAAAGGAACAATGC-3′ and 5′-GTGCACAGCAAAGTGATTGG-3' (*Nox2*), 5′-TGCAGAGATATCCAGTCCTTCC -3′ and 5′-TCCCATCTGTTTGACTGAGG-3' (*Nox4*), [3], 5′- CTTGAACTCTATGGCGGGTTC-3′ and 5′-GCCATCCTGCTTCCAAAGTC-3’ (*Ascl1*), 5′-ATAGACCCCAGCGGCAACTA-3′ and 5′-CCGAACACTGTCCATGGTTC -3’ (*Tubb3*), 5′-ATGCAGTTGTCCCTCCATTC-3′ and 5′-ATGCCACCAAGTTGTCATCA-3’ (*Dcx*), 5′- AAGATGAAACCAACCTGAGGC-3′ and 5′-CGAACTTCCTCCTCATAGATC -3’ (*Gfap*), 5′-GGGAATGGTGAAGACCGTGT-3′ and 5′-CCGCATGGACTGTGGTCATGA-3′ (*cFos*), 5′-CACTCTCCCGTGAAGCCATT-3′ and 5′-TCCTCCTCAGCGTCCACATA-3′ (*Arc*), and 5′-TCAGCAATGCCTCCTGCACCA-3′ and 5′-GCATGGACTGTGGTCATGAG -3’ (*Gapdh*). Relative mRNA levels were normalized to *Gapdh* abundance from the same sample and expressed as fold *versus* the corresponding normalized values in WT NPC.

*Immunocytochemistry.* Neurons or neurospheres on glass coverslips were fixed with 4 % (w/v) (in PBS) paraformaldehyde in PBS for 30 min and immunostained with anti-NRF2 (1/500; ab62352, Abcam), anti-BrdU (1:500; M#1406, Sigma-Aldrich), anti-MAP2 (1:500; ab11268, Abcam), and anti-TAU (1:500; ab8152, Abcam). Immunolabeling was detected using IgG-Cy2 (1:500) and IgG-Cy3 (1:500) secondary antibodies (1:500; Jackson ImmunoResearch). Nuclei were stained with 4′,6-diamidino-2-phenylindole (DAPI; D9542, Sigma-Aldrich). Coverslips were washed, mounted with SlowFade light antifade reagent (Invitrogen). For BrdU studies, cells were incubated with 10 μM Bromodeoxyuridine (BD Pharmingen™), for 30 min, and treated with 2 N HCl, for 30 min [28].

*Flow cytometry detection of cell death.* Neurospheres were dissociated and stained with annexin V–DY634 (Immunostep) and 7-aminoactinomycin D (7-AAD; Becton Dickinson Biosciences) in binding buffer (100 mM HEPES, 140 mM NaCl, and 2.5 mM CaCl_2_). NPC were analyzed, in three replicates per condition, on a FACScalibur flow cytometer (15-mW argon ion laser tuned at 488 nm; CellQuest software, Becton Dickinson Biosciences) to quantitatively determine the percentage of apoptotic neurons. The annexin V–DY634–stained neurons that were 7-AAD negative were apoptotic. Data were expressed as percentages [33].

*Cell cycle analysis by flow cytometry.* Neurospheres were dissociated and fixed with cold 70 % ethanol for 3 min at 4 °C. NPC were washed with a buffer containing Tween in PBS and incubated with propidium iodide solution (Sigma-Aldrich), for 20 min at 37 °C. Samples were then analyzed in the FL3 channel using a FACScalibur flow cytometer.

*Telomere Length.* Telomere length was measured by qPCR using the commercial Absolute Mouse Telomere Length Quantification qPCR Assay Kit (ScienCell, Catalog #M8918), following manufacturer's instructions. The amplification reaction was carried out using a Mastercycler® ep Realplex thermocycler (Eppendorf) and the Applied Biosystems StepOnePlus™ Real-Time PCR System. Fragments were amplified using 1 μL of each specific oligonucleotide, 10 μL of SYBR Green PCR MasterMix (Applied Biosystems), and 7 μL of water. The total amount of DNA in each reaction was 5 ng. The amplification program was as follows: 10 min at 95 °C, followed by 40 cycles of 95 °C for 15 s and 60 °C for 1 min. The comparative Ct method [2−ΔΔCt] was used to calculate the relative expression of each amplicon and to quantify the results.

*NADPH(H*^*+*^*)/NADP* + *ratio determination.* This was determined with a colorimetric NADP^+^/NADPH assay kit (Abcam). Cells were re-suspended in 500 μl extraction buffer, vortexed and centrifuged at 14,000×*g* for 5 min to remove insoluble material. The supernatant was used for NADPH(H^+^) plus NADP^+^ measurement. NADPH(H^+^) was determined in 200 μl of the supernatant after being heated at 60 °C for 30 min to decompose NADP^+^. The actual NADP^+^ and NADPH(H^+^) concentrations were calculated by extrapolation of the values to a NADPH(H^+^) standard curve (0–100 pmol per well).

*NADH(H*^*+*^*)/NAD* + *levels.* We used a fluorimetric NAD^+^/NADH(H^+^) assay kit (MAK460; Sigma-Aldrich). Neurospheres (∼10^6^ cells) were centrifuged and homogenized in 100 μL extraction buffer and heated at 60 °C, for 5 min. Following instructions, a 1:1 volume of the opposite extraction buffer was added, followed by 20 μL of assay buffer, mixed, and centrifuged at 14,000×*g* for 5 min. The supernatant was transferred to 96-well opaque flat-bottom plates (50 μL/well). For the 2X reaction mix, each sample received 40 μL assay buffer, 1 μL enzyme A, 1 μL enzyme B, 10 μL lactate, and 5 μL probe. A 50 μL reaction mix was added to each 50 μL sample, mixed, and fluorescence was measured at 530 nm excitation/585 nm emission at time 0 using a Varioskan Flash (Thermo Scientific). After a 10-min incubation in the dark, the measurement was repeated. NADH(H^+^)/NAD^+^ concentrations were determined using a standard curve ranging from 0 to 1 μM NAD^+^.

*Determination of metabolic fluxes.* To assess glycolysis and pentose-phosphate pathway (PPP), we used radioisotopic approaches. Neurospheres were disgregated with Accutase™ and seeded in 25 cm^2^ flasks hanging a microcentrifuge tube containing either 1 ml benzethonium hydroxide (Sigma) (for ^14^CO_2_ equilibration) or 1 ml H_2_O (for ^3^H_2_O equilibration). All incubations were carried out in KRPG, containing 5 mM d-glucose at 37 °C in the air-thermostated chamber of an orbital shaker. To measure the carbon flux from glucose to CO_2_, cells were incubated in KRPG (5 mM glucose) buffer with 0.3 μCi/ml of [6–^14^C]- or [1–^14^C]-glucose. Incubations were terminated after 90 min by the addition of 0.2 ml 20 % perchloric acid (Merck Millipore) and, after a further 30 min, the tube containing benzethonium hydroxide (with the trapped ^14^CO_2_) was used to determine the radioactivity using a liquid scintillation analyzer (Tri-Carb 4810 TR, PerkinElmer). Glucose flux through the PPP was estimated by subtracting the [6–14C]-glucose flux from the [1–14C]-glucose flux. The glycolytic flux was measured by assaying the rate of ^3^H_2_O production from [3-^3^H] glucose using a similar strategy using 2 μCi/ml of D-[3-^3^H] glucose in KRPG buffer (5 mM d-glucose) for 180 min. After incubations were terminated with 0.2 ml 20 % perchloric acid, the cells were further incubated for 72 h to allow for ^3^H_2_O equilibration with H_2_O present in the central microcentrifuge tube. The ^3^H_2_O was then measured by liquid scintillation counting (Tri-Carb 4810 TR, PerkinElmer). The specific radioactivity was used for the calculations. Under these experimental conditions, 70 % of the produced ^14^CO_2_ and 28 % of the produced ^3^H_2_O were recovered and considered for the calculations [3].

*Neurospheres differentiation assay.* Neurospheres were cultured for 6 days in proliferation medium with growth factors (P). NPC were plated at a density of 10^5^ cells/cm^2^ on an adhesive substrate (Geltrex, Growth Factor Reduced Matrigel Matrix, Thermo Fisher) in DMEM/F12 medium, supplemented with 10 ng/ml of bFGF (basic Fibroblast Growth Factor) for 2 days (2). Mitogens were then removed, and cells were grown in medium supplemented with 2 % fetal bovine serum. Differentiating cultures were analyzed 24 h (2 + 1) and 3 days (2 + 3) after mitogen withdrawal [34].

*NPC migration assay.* NPC were cultured for 6 days in proliferation medium with growth factors. Neurospheres were then placed on glass coverslips coated with poly-d-lysine (10 μg/ml) and fibronectin (1 μg/ml), where cells migrate out of the neurosphere edges, forming a radial migration area. Phase-contrast images were taken at different times and NPC migration distance (μm) and neurosphere diameters (μm) were measured using the ImageJ 1.48u4 software (NIH). NPC migration was expressed as the migration distance index, which was calculated by average NPC migration distance/neurosphere diameter [35].

*Mice perfusion and immunohistochemistry.* Timed murine embryos were obtained from pregnant dams according to animal care standards of the University of Salamanca. E12 and E15 heads were fixed with 4 % (w/v) paraformaldehyde and methanol (10 %) in 0.1 M phosphate buffer pH 7.4, for 7 days at 4 °C. E18 and P0 heads were anesthetized by i.p. injection of xylazine and ketamine (1:4) at 1 ml/kg body weight, and perfused intra-aortically with 0.9 % NaCl, followed by 5 ml/g body weight of 4 % (w/v) paraformaldehyde in 0.1 M phosphate buffer (PB), pH 7.4. Brains were postfixed in 4 % PFA, overnight at 4 °C. Tissue blocks were rinsed in 0.1 M PB and sequentially immersed in 10 %, 20 % and 30 % (w/v) sucrose in PB until they sank. After cryoprotection (Tissue-Tek® O.C.T ™ Compound. Sakura) brain blocks were serially sectioned (20 or 40 μm-thick) with a freezing-sliding cryostate (Leica; CM1950 AgProtect). For immunohistochemistry, slices were rinsed in 0.1 M PB and then incubated sequentially in (i) 0.1 M ammonium chloride in PB for 30 min (to remove aldehyde autofluorescence); (ii) three PB washes of 10 min each; (iii) anti-MAP2 (1:1000; ab11268; Abcam), anti-NESTIN (1:500; 556309, BD), anti-BrdU (1:500; ab8152, Abcam), anti-TUJ1 (1:500; ab18207, Abcam), anti-TBR2 (1:500; ab216870, Abcam), anti TBR1 (1:1000; ab31940, Abcam), anti-SATB2 (1:50; ab92446, Abcam) and anti-CTIP2 (1:1000; ab18465, Abcam) in 0.2 % Triton X-100 (Sigma-Aldrich) and 10 % goat serum (Jackson Immuno-Research #005-000-121) in 0.1 M PB for 72 h, at 4 °C. For BrdU staining, sections were pre-treated with 2 N HCl at 37 °C for 30 min; (iv) three PB washes of 10 min each again; (v) fluorophore conjugated secondary antibodies, 1:500 Cy2 goat anti-mouse, Cy2 goat anti-rabbit, Cy3 goat anti-mouse or Cy3 goat anti-rabbit (1:500; Jackson Immuno-Research) in PB for 2 h at room temperature; and (vi) 0.5 μg/ml DAPI in PB for 10 min at room temperature. After being rinsed with PB, sections were mounted with Fluoromount (Sigma-Aldrich #F4680) aqueous mounting medium and microscope coverslips (Menzel, Thermo) [28].

*Imaging and quantification.* Samples were examined with different microscopes depending on staining: epifluorescence inverted microscope (Nikon; Eclipse Ti-E) with B/W CCD digital camera (Hamamatsu; ORCA-E.R.); Olympus IX81 Spinning disk confocal microscope (Olympus) with Evolve digital camera (Photometrics); white field inverted microscope (Leica; DMIRB) with digital camera (Nikon; Digital Sight) and Andor Dragonfly Spinning Disk confocal microscope (Oxford Instruments) equipped with SONA camera and Fusion 2.4.0.14 (Oxford Instruments). The nuclei of total cells were outlined and counted by using “analyze particles” tool, from ImageJ 1.48u4 software (NIH), creating a mask in which the BrdU^+^, pH3^+^, TBR1^+^, TBR2^+^, CTIP2^+^, SATB2^+^ cells were identified, and results were presented as percentage of positive cells within total cells. Nrf2 staining, the analysis was analyzed by mean density of nuclear fluorescence. MAP2 and TAU was analyzed by % Area of staining using ImageJ 1.48u4 software (NIH) always applying “auto” function.

*Statistical analysis.* Statistical results were expressed as mean ± standard error of the mean (S.E.M.). Samples independently used per experiment are indicated in figure legends. Previous studies have been used to estimate the sample size. All samples, animals and data points were included in the analyses. Datasets were first subjected to normality (Shapiro-Wilk test) and equal variances tests (Levene's test). For simple comparison of parametric sample unpaired two-tailed Student's *t*-test or Welch's *t*-*t*est were used, and for non-parametric datasets Unpaired U Mann-Whitney's test were applied. Of parametric samples we used one-way ANOVA or Welch's ANOVA followed by its respective post hoc tests (Bonferroni or Games-Howell) and for non-parametric samples we applied Kruskal-Wallis test followed by Dunn's test correction. All statistical tests used are specified in the figure legends. Statistical analyses were performed using GraphPad Prism v8 software and IBM© SPSS® Statistics v27.

## Funding source

This work was funded by 10.13039/501100004587Instituto de Salud Carlos III, co-funded by the 10.13039/501100000780European Union, (PI21/00727, PI24/00810, RD24/0009/0005 to AA; CB16/10/00282 to JPB), and funded by the European Union - NextGeneration EU, Recovery and Resilience Facility (RD21/0006/0005, PMP22/00084 to AA), Junta de Castilla y León (CSI011P23 and Escalera de Excelencia CLU-2017-03 to AA and JPB), Agencia Estatal de Investigación (MICIU) (PID2022-138813OB-I00 and 10.13039/501100002924FEDER, 10.13039/501100012637UE to JPB; RED2022-134407-T to AA), and 10.13039/501100019179la Caixa Foundation (grant agreement LCF/PR/HR23/52430016 to JPB), the European Union's 10.13039/100018693Horizon Europe research and innovation program under the MSCA Doctoral Networks 2021 (101072759; FuEl ThEbRaiN In healtThY aging and age-related diseases, ETERNITY, to A.A. and J.P.B.), and the 10.13039/501100000781European Research Council (ERC) Advanced Grant NeuroSTARS (ref. 101199747, to J.P.B.)

## CRediT authorship contribution statement

**Regina Mengual:** Data curation, Formal analysis, Investigation, Methodology, Writing – original draft, Writing – review & editing. **Verónica Bobo-Jiménez:** Data curation, Formal analysis, Investigation, Methodology, Writing – review & editing. **Cristina Rodríguez:** Investigation, Methodology, Writing – review & editing. **Rebeca Lapresa:** Formal analysis, Investigation, Writing – review & editing. **Darío García-Rodríguez:** Investigation, Writing – review & editing. **Daniel Jiménez-Blasco:** Investigation, Writing – review & editing. **Elisa Cabiscol:** Investigation, Writing – review & editing. **Joaquim Ros:** Investigation, Writing – review & editing. **María Delgado-Esteban:** Formal analysis, Investigation, Methodology, Supervision, Writing – review & editing. **Juan P. Bolaños:** Data curation, Formal analysis, Methodology, Resources, Supervision, Writing – original draft, Writing – review & editing. **Ángeles Almeida:** Conceptualization, Data curation, Formal analysis, Funding acquisition, Investigation, Methodology, Resources, Supervision, Writing – original draft, Writing – review & editing.

## Declaration of competing interest

none.

## Data Availability

Data will be made available on request.
